# Role of Rotifers in Betanodavirus Transmission to European Sea Bass Larvae

**DOI:** 10.3389/fvets.2022.932327

**Published:** 2022-08-03

**Authors:** Lucia Vázquez-Salgado, Francesco Pascoli, Andrea Marsella, Lorena Biasini, Alessandra Buratin, Tobia Pretto, Miriam Abbadi, Erica Melchiotti, Isabel Bandín, Anna Toffan

**Affiliations:** ^1^Departamento de Microbiología y Parasitología, Instituto de Acuicultura, Universidade de Santiago de Compostela, Santiago de Compostela, Spain; ^2^Istituto Zooprofilattico Sperimentale delle Venezie, OIE Reference Laboratory for Viral Encephalo-Retinopathy, National Reference Laboratory for Fish Diseases Legnaro, Padova, Italy; ^3^Department of Histopathology, Istituto Zooprofilattico Sperimentale delle Venezie Legnaro, Padova, Italy

**Keywords:** rotifer (*Brachionus plicatilis*), vector, sea bass larvae, nodavirus (NNV), RGNNV, transmission, oral infection

## Abstract

Marine invertebrates such as rotifers or *Artemia*, frequently used for fish larvae feeding, can be a potential source of pathogens. It has been demonstrated that *Artemia* can act as a nervous necrosis virus (NNV)-vector to Senegalese sole larvae. Therefore, in this study, we aimed to clarify the role of rotifers in NNV transmission to sea bass larvae following an oral challenge. Our results showed that sea bass larvae fed on a single dose of rotifers retaining NNV displayed clinical signs, mortality, and viral replication similar to the immersion challenge, although the course of the infection was slightly different between the two infection routes. Furthermore, we also demonstrated that rotifers can internalize NNV particles due to their filtering nature and maintain virus viability since viral particles were detected by immunohistochemistry, immunofluorescence, and cell culture within the rotifer body. However, viral quantification data suggested that rotifers are not permissive to NNV replication. In conclusion, this research demonstrated NNV horizontal transmission through rotifers to sea bass larvae, highlighting the importance of establishing strict routine controls on live food to prevent the introduction of potential pathogens to hatcheries.

## Introduction

Viral encephalopathy and retinopathy (VER) is a neurological pathology of fish caused by the nervous necrosis virus (NNV; G. *Betanodavirus*, F. *Nodaviridae*) that provokes high mortalities, especially in the early stages of development, and leads to great economic losses in the farming industry throughout the world (1). The viral genome is composed of two molecules of (+) ssRNA, known as RNA1 and RNA2, encoding the viral polymerase (RdRp) and the coat protein, respectively (2). A third RNA molecule is synthesized during the infection process, namely, RNA3, and encodes two non-structural proteins that favor viral replication by suppressing cell apoptosis and silencing interfering RNAs (3, 4). The RNA2 segment contains the T4 region, which enables the classification of the NNV into four genotypes, namely, barfin flounder (BFNNV), red-spotted grouper (RGNNV), striped jack (SJNNV), and tiger puffer nervous necrosis virus (TPNNV) (5). In addition, natural reassortants containing RNA1 and RNA2 typed as RGNNV or SJNNV (RGNNV/SJNNV and SJNNV/RGNNV) have been reported in farmed and wild fish from southern Europe (6–9).

Betanodavirus transmission in nature can occur horizontally or vertically (1). The horizontal route involves transmission through the water column or by contact between infected or asymptomatic carriers with susceptible healthy animals (10). Wild marine invertebrates as well as some zooplankton species including the crustacean *Artemia salina* or the rotifer *Brachionus plicatilis*, which are commonly used as starting food for marine larvae, have shown to be NNV reservoirs or vectors (11–18). In fact, a reassortant NNV strain was isolated from the brine shrimp *Artemia salina* (8), and this crustacean was shown to effectively transmit NNV infective particles to Senegalese sole (*Solea senegalensis*) larvae causing disease signs and high mortality (19). However, there is no evidence of the role of rotifers in NNV transmission to fish larvae. This study aimed to assess NNV transmission through rotifers to European sea bass (*Dicentrarchus labrax*) larvae, one of the most farmed fish species in Mediterranean aquaculture.

## Methodology

### Viral Inoculum Production

The RGNNV-type strain 283.2009 isolated in Italy from farmed European sea bass, showing mass mortalities (8), was used in this study. The viral strain was propagated in E-11 cell monolayers, a cell clone derived from SSN-1 cell line (20), grown for 24 h in 75 cm^2^ tissue culture flasks with the Minimum Essential Medium (MEM) containing 10% fetal bovine serum (FBS), 1% glutamine, and 1% antibiotics (penicillin and streptomycin). Prior to viral inoculation, the cell culture medium was replaced with MEM FBS-free and incubated at 25°C. When the cytopathic effect (CPE) was extended, the viral suspension was collected and centrifuged at 3,000 *g* for 15 min. Clarified virus suspension was stored at −80°C until use. Viral titer was calculated in quadruplicate in SSN-1 monolayers (striped snakehead, *Ophicephalus striatus*) using the end point procedure (21), and titers were expressed as TCID_50_/ml.

### Rotifer NNV-Exposure Assay

Rotifers (*Brachionus plicatilis*) were grown under continuous culture in 80-l opaque circular tanks with artificial seawater (salinity 37 PSU) at 27 ± 1°C and aeration. Cultures were fed daily with the NannoStar rotifer diet (AlgaSpring, The Netherlands), a commercial product containing concentrated *Nannochloropsis* adequate for a correct rotifer performance.

This assay was performed using one rotifer batch (~10^6^ rotifers) challenged by bath immersion with the 283.2009 strain (10^5^ TCID_50_/ml) in a 20-l tank for 24 h. Later, rotifers were filtered and rinsed with tap water and cultivated for an additional 6 days in virus-free water under previously described conditions. Rotifer samples (200 mg) were collected at 6, 12, 24, 48, 72, and 144 h post-challenge (hpc) and rinsed prior to performing the analysis. NNV-exposed rotifers were tested using the indirect fluorescence antibody test (IFAT) at 6, 12, and 24 h post-challenge and immunohistochemistry (IHC) at 6, 12, 24, and 48 hpc, while two-step quantitative real-time PCR (RT-qPCR) and virus isolation in cell culture were applied to challenged rotifers at all time points as described in the following sections. A 20-ml water aliquot was also collected from the tank at 10 min post-challenge in order to confirm the NNV dose.

A mock-challenged rotifer batch was also set up, handled, and tested as the NNV-exposed group.

### Sea Bass Larvae Infection

European sea bass larvae (0 days post-hatch, *n* = 60,000) were purchased from a NNV-free commercial fish farm and housed at the experimental aquarium facilities of the Istituto Zooprofilattico Sperimentale delle Venezie (IZSVe, Italy). Prior to performing the experimental infections, a 10 mg sample of larvae was analyzed and tested negative for the most common sea bass pathogens (bacteria and betanodavirus). Larvae were equally distributed in three opaque 300-l tanks (20,000 larvae per tank), filled with artificial salt water (salinity 35–27 PSU), and the temperature was maintained at 19 ± 1°C and oxygen at 6 ± 0.5 ppm. Larvae were kept in darkness until the adsorption of the yolk sac (~9 days post-hatching), and later, they were maintained with an artificial photoperiod of 7 h of light and at 18°C. In addition, prior to the infection trials, larvae were fed for 3 days with *Nannochloropsis*-enriched rotifers to ensure an appropriate food intake.

Sea bass larvae were challenged at 12 days post-hatching (dph). The oral infection was carried out by feeding larvae from 1 tank (*n* = 20,000) with a single dose of NNV retaining rotifers (~100 rotifers/larvae), as previously described in the “Rotifer NNV-exposure assay” section, for 18 h with the 283.2009 strain at 10^6^ TCID_50_/ml. NNV-exposed rotifers were rinsed carefully under tap water and concentrated in 20-l before their administration as feed to sea bass larvae. For comparative purposes, the remaining larvae were bath and mock-challenged for 1 h with the 283.2009 strain (10^5^ TCID_50_/ml) and MEM, respectively. Fish samples were collected daily from each of the three tanks for viral load quantification by RT-qPCR, titration in cell culture, and IHC. Larvae were also monitored daily for signs of disease.

### Sample Processing

#### Viral Load Assessment by Titration (TCID_50_)

Rotifer (200 mg) and larvae (40 larvae/time point) samples were manually homogenized in tubes containing sterile quartz sand and diluted in 500 and 330 μl of MEM, respectively. Then, homogenates were clarified by centrifugation for 2 min at 11,000 *g*, and the harvested supernatants were incubated overnight at 4°C with 1% antibiotic and antimycotic solution (10,000 IU/ml penicillin G, 10 mg/ml streptomycin sulfate, 25 μg/ml amphotericin B, 0.4% of 50 mg/ml kanamycin solution, and 1% of 50 mg/ml solution of polymyxin B sulfate) (Merck KGaA, Darmstadt, Germany).

The water aliquot was filtered using a 0.45 μm membrane and incubated overnight as previously described.

Samples were titrated on semiconfluent SSN-1 monolayers as detailed in the “Viral Inoculum Production” section.

The samples that showed the absence of CPE, corresponding to a viral titer below the limit of detection of the cell culture titration, was given the arbitrary value of 10^0^ TCID_50_/ml.

#### RNA1 Quantification by RT-qPCR

Rotifer (triplicate of 20 mg each) and pooled larvae (*n* = 15; triplicate pools of 5 larvae each) samples were homogenized with stainless steel beads in a TissueLyser II (Qiagen, Hilden, Germany). The total RNA was extracted from homogenized rotifers, larvae samples, and the water aliquot using the RNeasy Mini Kit (Qiagen, Hilden, Germany) following the manufacturer's instructions. RNA integrity was verified in an Agilent 2100 Bioanalyzer System using an Agilent RNA 6000 Nano Kit (Agilent Technologies, Santa Clara, USA), and concentration was estimated using the Qubit™ RNA BR Assay kit in a Qubit™ 4 Fluorometer (ThermoFisher Scientific, Waltham, USA). The RNA amount of all samples was normalized in molecular-grade water prior to performing the reverse transcription. For the RNAs of rotifers and water, the normalization was carried out based on the starting weight and volume of samples, respectively, assuming a 100% RNA extraction yield. The larvae RNA normalization, however, was based on the concentration of total RNA extracted. RNA reverse transcription was performed using a SuperScript™ III Reverse Transcriptase Kit (Invitrogen by Thermofisher Scientific, Waltham, USA). In brief, complementary DNA (cDNA) was synthesized starting from normalized RNA equivalent to 400 μg of rotifer, 4 μl of water, and 80 ng of total larvae RNA. The reaction was performed in 20 μl final volume using 50 pmol of random hexamers and 10 mmol of dNTPs. The thermal profile consisted of a preincubation step for 5 min at 65°C followed by incubation on ice for at least 5 min, then 5 min at 25°C, 1 h at 50°C, and 15 min at 70°C. Real-time PCR, targeting the RNA1 molecule, was performed in 25 μl final volume using the SsoFast™ EvaGreen^®^ Supermix (BioRad, Hercules, USA), 5 μl of obtained cDNA, and 0.5 μM of each forward and reverse primer set (RNA1_FOR 5′-ATCACTGACGACTCCGTTCACTACCG-3′ and RNA1_REV 5′-CATACATGGTATCCTGGTTGTAGTTCC-3′). RT-qPCR reactions were performed in a CFX96™ Real-Time System (BioRad, Hercules, USA) and the amplification thermal profile consisted of an activation step of 30 s at 95°C followed by 40 cycles of denaturation (5 s at 95°C) and an annealing-extension step (5 s at 60°C). The reactions were terminated with a final melting temperature curve analysis from 65 to 95°C (5 s/step, ramp rate 0.5°C/s). Absolute RNA1 quantification was estimated by comparing obtained quantification cycle (Cq) values with standard curves prepared using 6 dilutions (10^7^-10^2^ copy numbers/reaction) of synthetic RNA1 tested in triplicate. Synthetic RNA1 was produced according to Toffan et al. (22). Viral quantification was expressed as logarithmic values of RNA1 copy number (LCN) detected in 100 μg (rotifers), 1 μl (water), or 20 ng of total RNA (larvae). Hereafter, the reported LCN values refer to the mean of the 3 analyzed biological replicates. To give better representativeness of the entire population, LCN values below the limit of quantification (LoQ) were also included in the analysis, even though considered numerically less reliable. The undetected samples were given the arbitrary value of 0 LCN.

### Rotifer IFAT

Rotifer samples (6, 12, and 24 hpc) were preserved and fixed in buffered formalin 4% (VWR International Srl, Italy) at 4°C until use. Prior to performing the IFAT, samples were washed with PBS-Tween (0.05%) and blocked with 0.05% (w/v) bovine serum albumin (BSA) for 30 min at 37°C. First, rotifers were incubated with an anti-RGNNV polyclonal serum (1:1,000) produced in-house (pAb 283) (23) and then with FITC conjugated goat anti-rabbit serum (1:100) (Merck, Darmstadt, Germany). Both incubations were performed for 1 h at 37°C. Fluorescence was observed in a Zeiss Axioskop microscope equipped with an Axiocam MRC 5.

### Rotifer and Larvae IHC Staining

Rotifer samples were collected at 6, 12, 24, and 48 hpc, while the mock-challenged rotifer control was collected at 24 h.

Samples from sea bass larvae (*n* = 20) and rotifers were fixed in 10% buffered formalin and processed for histopathological and IHC examination (22). In brief, samples were dehydrated through a graded ethanol-xylene series and embedded in paraffin. Sections of 3 μm were first deparaffinized, rehydrated, and then stained with hematoxylin-eosin (H&E) for histopathological examination. IHC was performed automatically from de-waxing to counter-staining using the Discovery-Ultra instrument (Ventana Medical Systems Inc., Tucson, USA).

Following antigen retrieval with Protease 2 (Ventana Medical Systems Inc., Tucson, USA) for 32 min at 36°C, the IHC reaction was performed with rabbit anti-RGNNV polyclonal serum produced in-house (pAb 283) (23), at the dilution of 1:5,000 in antibody diluent (Ventana Medical Systems Inc., Tucson, USA) for 1 h at room temperature. Samples were incubated with the secondary antibody and conjugated with alkaline phosphatase UltraMap Rabbit AP for 16 min and Chromogen Discovery Red for 32 min (Ventana Medical Systems Inc., Tucson, USA). Slides were then counterstained with Mayer's hematoxylin for 8 min, air-dried, dipped in xylene, and mounted in Eukitt medium (Kaltek, Saonara, Italy). The presence of betanodavirus antigens was characterized by bright red color.

### Statistical Analysis

Statistical tests were conducted using GraphPad Prism 8.0 (San Diego, California, USA). LCN data represent the mean of 3 replicates per time point. The dataset was first checked for normality using the Shapiro-Wilk test. Significant differences in viral loads between the two infection routes were assessed using two-way ANOVA and Sidak's correction, and *p* < 0.05 indicates statistically significant differences.

## Results

### Rotifer Challenge

#### Viral Load: TCID_50_ and LCN

**Figure 1:**
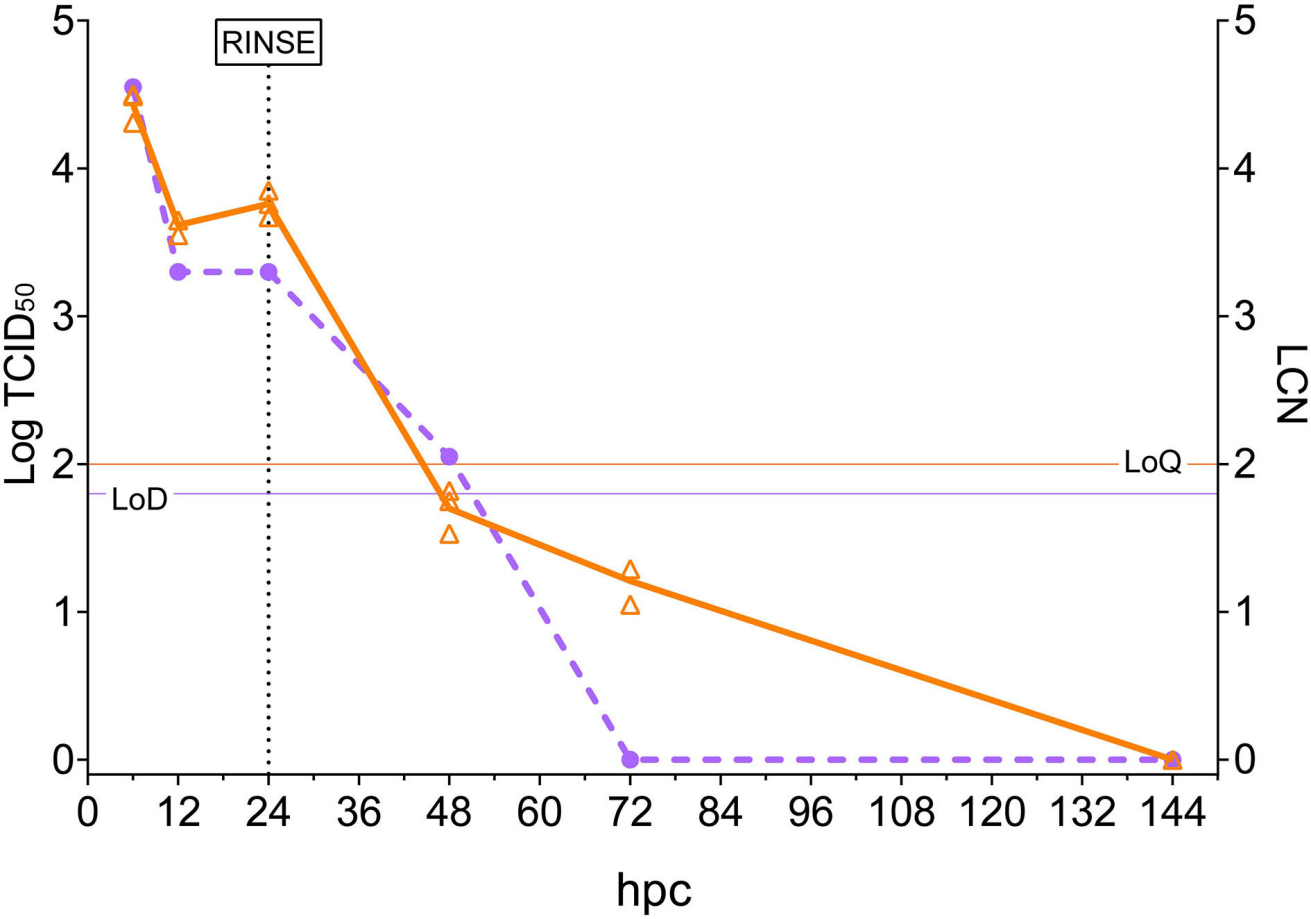
Rotifer nervous necrosis virus (NNV) challenge. The *x*-axis represents the observation period expressed as hours post-challenge (hpc). The left *y*-axis reports quantified viral loads expressed as Log_10_ of TCID_50_/ml, and values are depicted as circles. A dashed purple line shows the trend of TCID_50_/ml in time. The right *y*-axis reports quantified viral load expressed as LCN/100 μg, and values are depicted as triangles (three biological replicates for each sampling). A solid orange line shows the trend of mean LCN values in the time. Thin horizontal lines represent the limit of detection (LoD) or the limit of quantification (LoQ) of the methods. The vertical dashed line indicates the time in which NNV-exposed rotifers were rinsed and started their housing in NNV-free water.

Viral load quantification in rotifers was evaluated by observing both the viral titers and the LCN values over time (Figure 1). The highest values, in both viable particles and genomic load, were recorded at 6 hpc (10^4.55^ TCID_50_/ml and 4.43 LCN). At 12 hpc, a 1-log decrease was observed in the rotifer viral load (10^3.30^ TCID_50_/ml and 3.62 LCN) and remained almost stable until 24 hpc. Then, rotifers were filtered, rinsed, and placed in virus-free water. Samples from the subsequent time points showed a rapid and continuous decrease in viral loads. In fact, while a low titer (10^2.05^ TCID_50_/ml) was recorded at 48 hpc, no viral titers were detectable at 72 and 144 hpc. The same trend was observable while looking at the RT-qPCR results at 48 and 72 hpc (1.70 and 1.21 LCN respectively), and viral RNA was undetectable at 144 hpc. Moreover, the water aliquot collected from the challenge tank at 10 min pc was analyzed and displayed values of 10^4.80^ TCID_50_/ml and 5.08 LCN. Such results confirmed the theoretical NNV challenge dose. Mock-challenged rotifers collected at 24 hpc tested negative with both methods (data not shown).

#### Indirect Fluorescence Antibody Test

**Figure 2:**
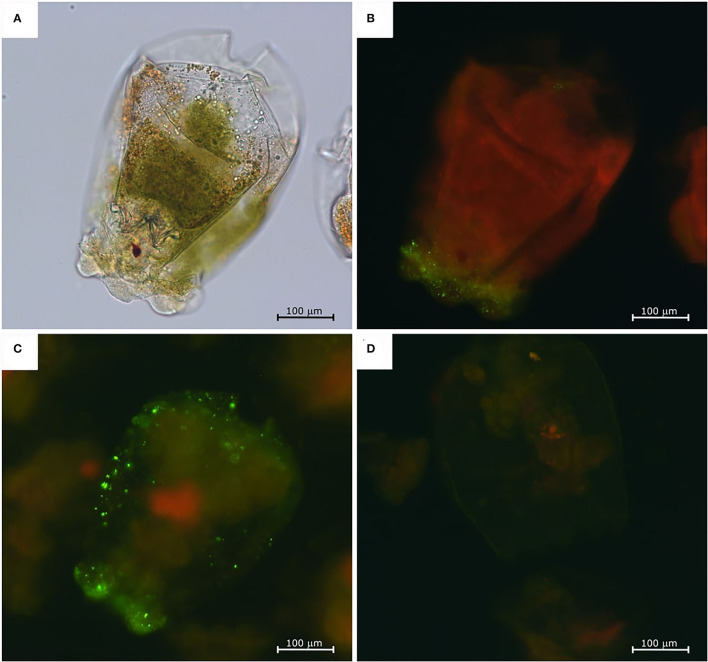
Nervous necrosis virus-exposed rotifers immunofluorescence assay. Challenged rotifer at 6 hpc, normal appearance under a light microscope **(A)**, and fluorescent microscope **(B)**. Fluorescence staining is visible mainly in the crown area. Challenged rotifer at 24 hpc **(C)**, and fluorescence localized on the crown and in the pseudocoelom. Negative control at 24 h **(D)**.

The NNV-challenged rotifers appeared normal in shape (Figure 2A) and did not show any abnormal behavior or changes in swimming capacity. The immunofluorescence assay showed a slight fluorescence in rotifers at 6 hpc (Figure 2B), but the strongest signals, with similar intensity, were detected at 12 and 24 hpc (Figure 2C). Fluorescence was mainly concentrated in the anterior part of the rotifer body, particularly in the corona, the organ responsible for the internalization of particles suspended in the water, and in the cuticle, the organ surrounding the rotifer body. The immunofluorescence signal was revealed neither at later time points nor in rotifer eggs nor in the mock-challenged rotifer batch (Figure 2D).

#### IHC Staining

**Figure 3:**
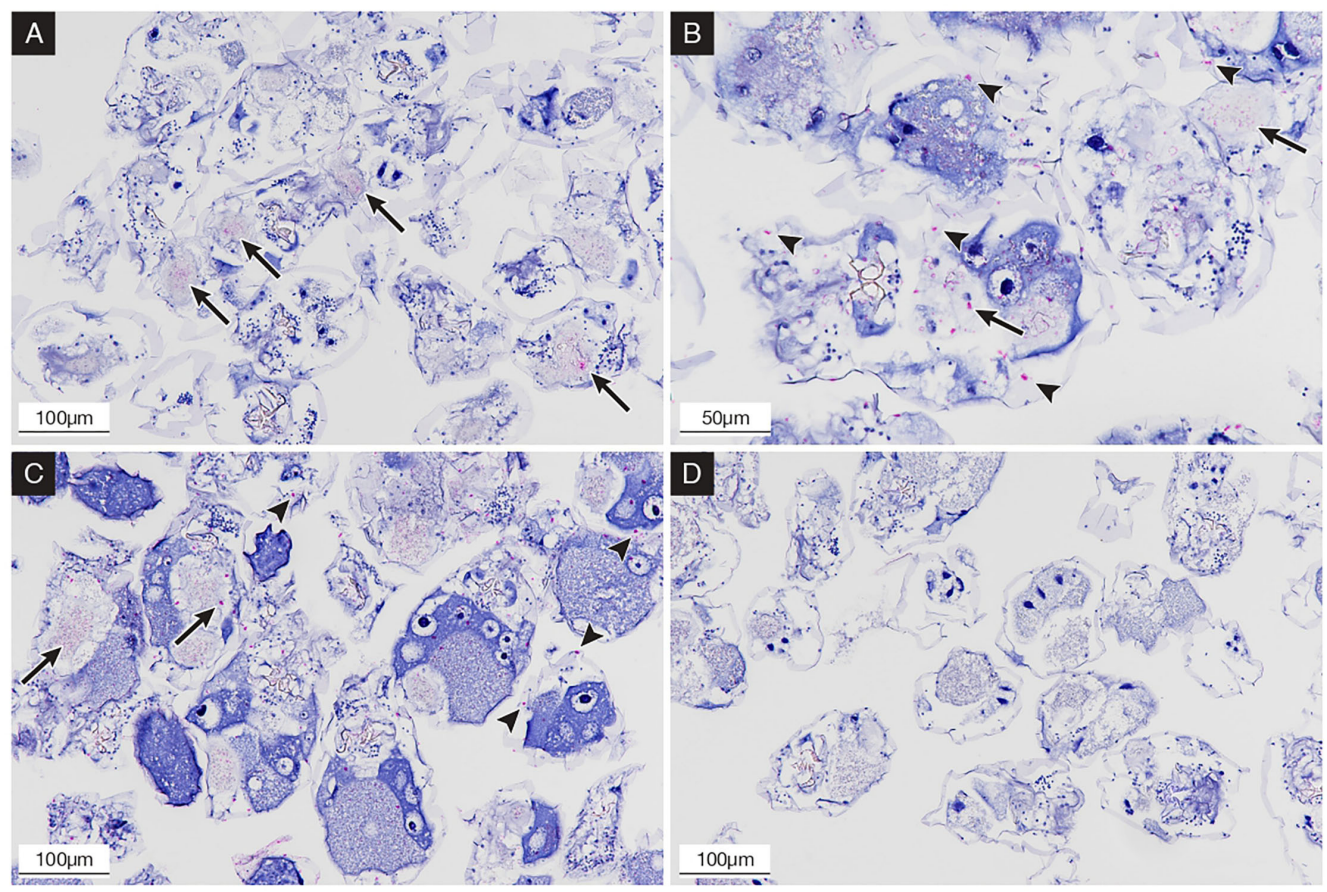
Immunohistochemical (IHC) staining of NNV-exposed rotifers at 6, 24, and 48 hpc; the presence of betanodavirus antigens was revealed in bright red color. **(A)** 6 hpc; rotifers showing IHC staining in the stomach and intestinal tract (arrows); **(B)** 24 hpc; rotifers showing IHC staining in the gut content (arrows) and in the pseudocoelom and inner cuticle (arrowheads); **(C)** 48 hpc; rotifers showing increased IHC staining in pseudocoelom (arrowheads); **(D)** mock-challenged rotifers after 24 h, negative control with no evidence of staining.

The IHC staining showed a positive signal at 6, 24, and 48 hpc. In detail, 283.2009-exposed rotifers initially evidenced the presence of IHC staining in the stomach and intestine lumen (Figure 3A) at 6 hpc, while at 24 (Figure 3B) and 48 hpc (Figure 3C), more IHC stainings were apparent in the pseudocoelom and in the inner side of the cuticle of NNV-challenged rotifers. No IHC signal was observed in the mock-challenged group (Figure 3D).

### Larvae Infection

Nervous necrosis virus-exposed rotifers showed a viral load of 10^4.55^ TCID_50_/ml (4.25 LCN) at the moment of feeding sea bass larvae, and bath water from the larvae challenge titrated 10^4.80^ TCID_50_/ml (4.63 LCN).

Oral infected larvae started to show common VER signs, consisting of abnormal swimming behavior, such as erratic swimming in circles or lying belly up, at 19 days postinfection (dpi), ending with the death of all individuals by 23 dpi. The bath infected group showed earlier disease signs (from 7 dpi), and no fish survived beyond day 12 pi. No VER signs were detected in the mock-challenged fish, and few losses were recorded during the challenge.

#### Viral Load Quantification by TCID_50_

**Figure 4:**
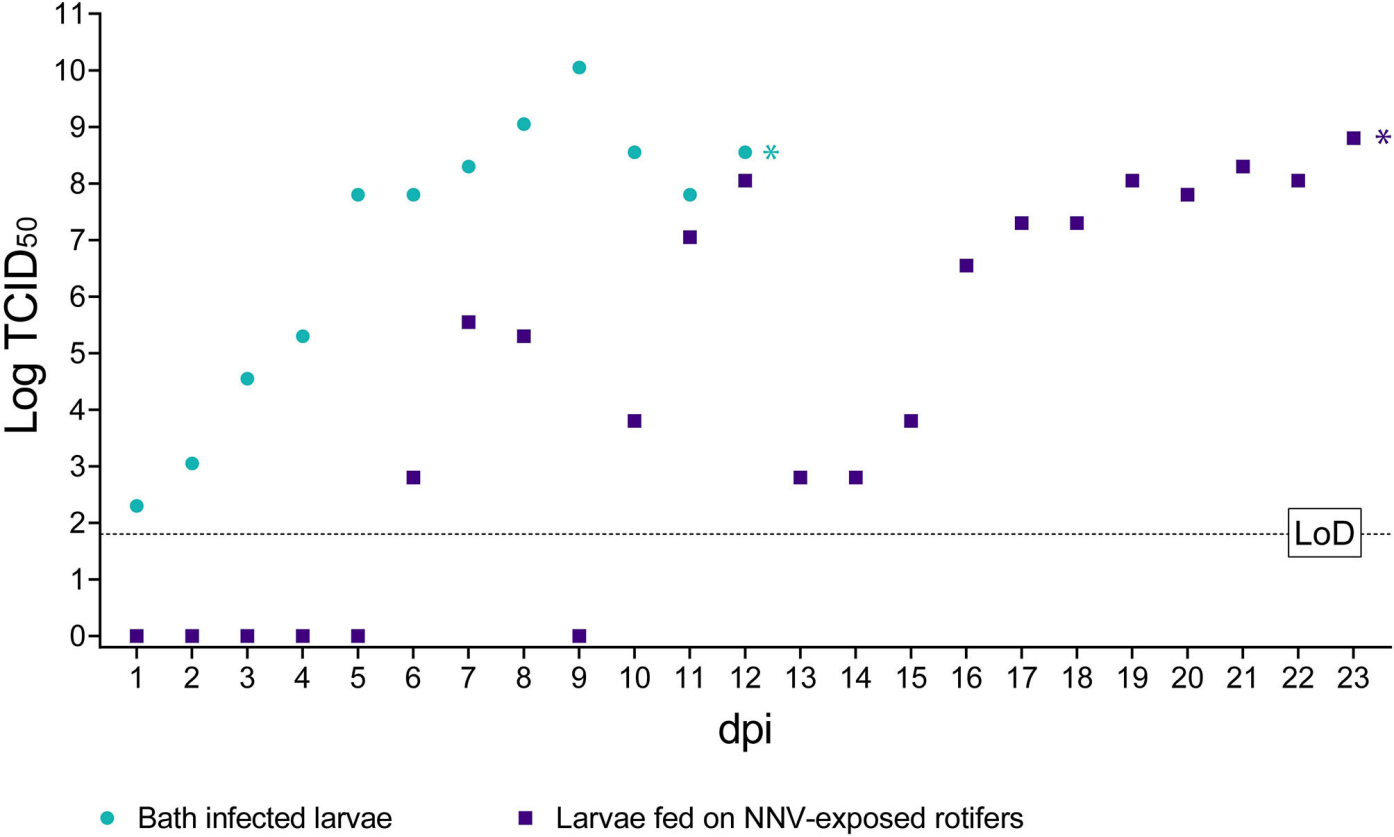
Infectious titer of NNV detected in challenged larvae. The *x*-axis represents the observation period expressed as days post-infection (dpi). The *y*-axis represents quantified viral load expressed as Log_10_ of TCID_50_/ml. Bath infected larvae values are depicted as green circles. Values of larvae fed on NNV-exposed rotifers are depicted as purple squares. A dashed horizontal line indicates the limit of detection (LoD). ^*^ Indicates the achievement of total mortality in both challenges.

No NNV viable particles were detected during the first 5 dpi in sea bass larvae fed with rotifers retaining NNV (Figure 4). Later on, the viral titers fluctuated considerably between days 6 and 12. However, from day 13 onward, titers progressively increased until the onset of clinical signs on day 19 pi, reaching a maximum value of 10^8.05^ TCID_50_/ml. Beyond this day and up to the end of the experiment, the viral loads remained almost the same, albeit with slight fluctuations (ranging from 10^7.80^ to 10^8.80^ TCID_50_/ml).

In larvae challenged by bath, the number of detected viable particles increased over time from 10^2.30^ TCID_50_/ml at 1 dpi to 10^10.05^ TCID_50_/ml on 9 dpi. Subsequently and until 12 dpi when all larvae were dead, the viral titers were maintained at such high values, ranging from 10^7.80^ to 10^8.55^ TCID_50_/ml (Figure 4). Mock-challenged sea bass larvae tested negative at all time points (data not shown).

#### Viral Load Quantification by RT-qPCR

RNA1 copy number quantification was evaluated in samples of pooled sea bass and analyzed in triplicates (5 larvae each) until the end of the experiment (12 dpi in the bath infected group and 23 dpi in larvae fed with rotifers retaining NNV).

**Figure 5:**
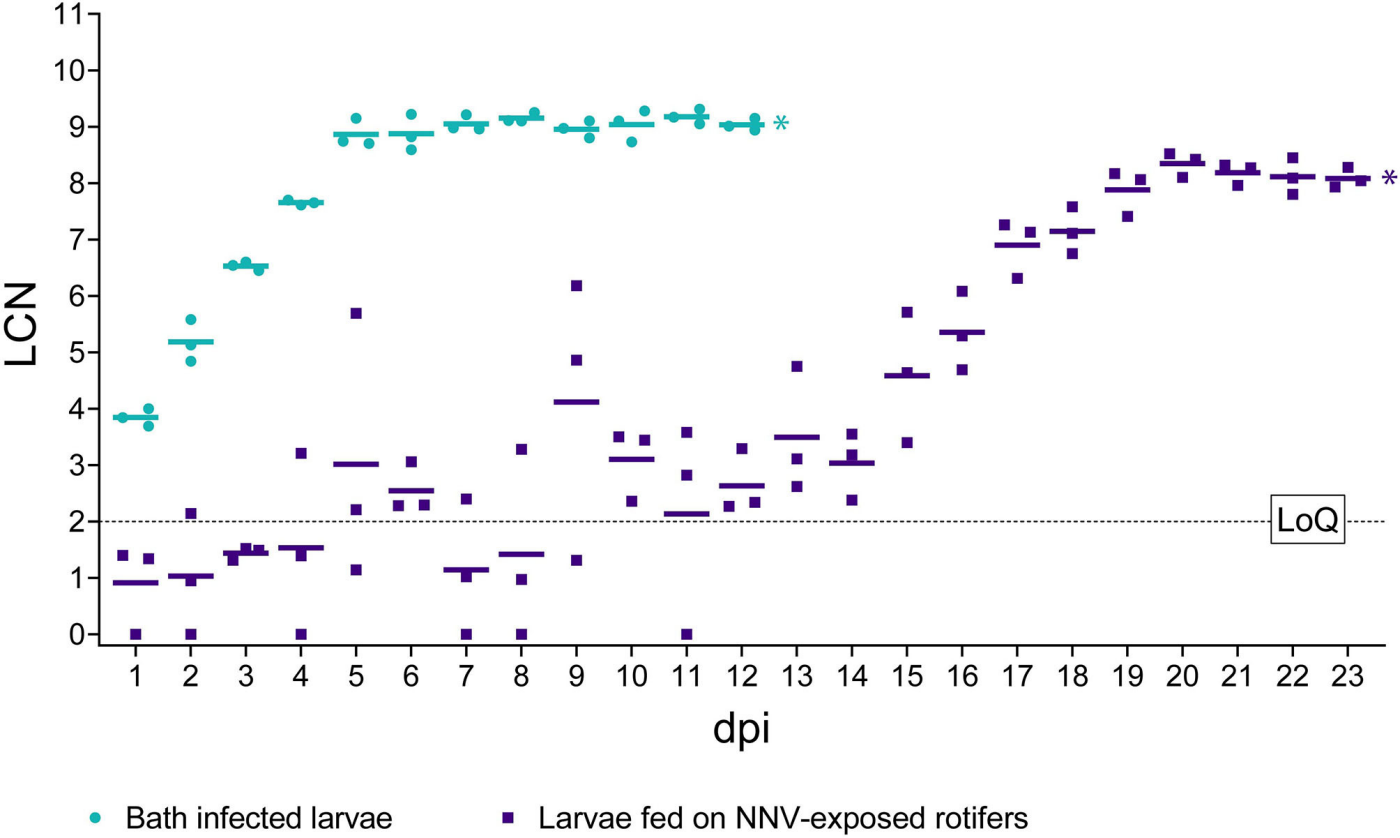
RT-qPCR results in challenged larvae. The *x*-axis represents the observation period expressed as days post-infection (dpi). The *y*-axis represents Log_10_ of RNA1 copy numbers (LCN) detected in 20 ng of total RNA. Results from bath infected larvae are depicted as green circles, while from larvae fed on NNV-exposed rotifers results are depicted as purple squares. Mean LCN values of the three replicates, per time point, are reported as short horizontal lines. A dashed horizontal line indicates the limit of quantification (LoQ). ^*^ Indicates the achievement of total mortality in both challenges.

Larvae orally challenged (Figure 5) showed average viral loads below 2 LCN until day 8 pi, and although on days 5 and 6 pi, the average number of RNA copies showed a slight increase (3.01 and 2.54 LCN, respectively). In general, a strong heterogenicity among biological replicates was observed, with standard deviation (SD) values higher than 1 in several sampling time points and reaching 2.51 at 9 dpi. From day 9 pi onward, a rise in the RNA copy number was observed although some oscillations until day 14. From this time point, an increasing trend in viral loads was detected until day 20 pi (mean LCN = 8.35 ± 0.22 SD), and a small SD among replicates was also observed from day 17 pi. Later, the number of genomic copies remained at similar values until the end of the experiment.

Bath challenged larvae showed very homogeneous values of LCN within biological triplicates and high values starting from 1 dpi (3.84 LCN ± 0.15 SD) (Figure 5). The number of NNV genomic copies increased regularly until day 5 pi (8.87 LCN ± 0.25 SD), with a further slight increase until 8 dpi, to plateau later. Details regarding individual LCN values are provided in Supplementary Material S2. Notably, no significant differences (*p* > 0.05) in the final viral load were observed between both infection routes. Mock-challenged sea bass larvae tested negative at all time points (data not shown).

#### Histological and IHC Analysis

**Figure 6:**
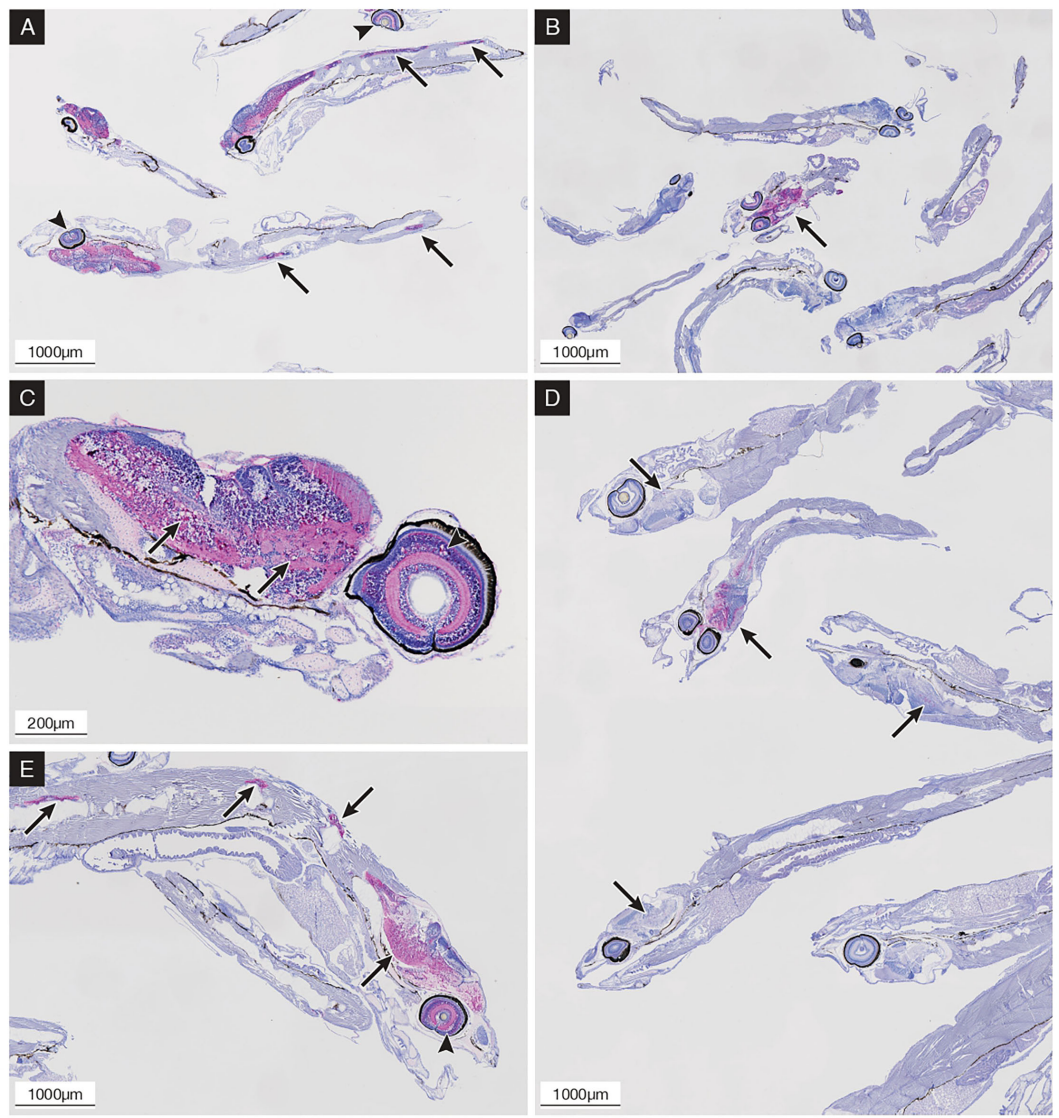
Immunohistochemical (IHC) staining of sea bass larvae challenged *via* the oral route **(B,D,E)** and by bath **(A,C)** at different time points; the presence of betanodavirus antigens was revealed in bright red color. **(A)** 9 dpi; bath challenged larvae showing diffuse central nervous system (CNS) and retina (arrowheads) IHC staining and CNS vacuolization; positive spinal cord highlighted with arrows. **(B)** 9 dpi; orally infected larvae showing single positive specimens with intense IHC staining in the CNS (arrow) and retina. **(C)** 10 dpi; bath challenged larvae showing diffuse IHC staining and vacuolization in the encephalon (arrows) and retina (arrowhead). **(D)** 17 dpi; orally infected larvae showing positive specimens with variable intensity of IHC staining in the encephalon (arrows). **(E)** 21 dpi; orally infected larvae showing diffuse IHC staining and vacuolization in the encephalon, spinal cord (arrows), and retina (arrowhead).

Oral infected larvae revealed the absence of IHC staining until 8 dpi when a single specimen (1+/13 larvae) showed the moderate presence of IHC staining in the encephalon, while the other larvae appeared negative. Only one specimen (1+/7) collected at 9 dpi showed intense IHC staining in the encephalon and retina with mild vacuolization (Figure 6B). Subsequently, no IHC positive specimens were observed until 15 dpi, followed by a progressive increase in the prevalence of positive larvae between 16 dpi and 19 dpi (Figure 6D), while after 20 dpi, all specimens appeared highly positive (details are provided in Supplementary Material S1A). With the exception of the early positive specimen at 9 dpi, vacuolization in the encephalon and spinal cord was increasingly observed after 16 dpi, while retinal vacuolization was recorded after 20 dpi (Figure 6E). Bath challenged larvae showed initial IHC staining in the central nervous system (CNS) starting from 3 dpi with a rapid increase in intensity and prevalence reaching 100% of positive larvae at 5 dpi, while IHC staining in the retina started at 5 dpi reaching 100% positive at 7 dpi. In the following sampling points, the prevalence and intensity of IHC staining (Figure 6A) remained the same until the end of the trial (day 12 pi) (details are provided in Supplementary Material S1B). Vacuolization of the CNS was noticeable from 6 dpi, whereas retinal vacuoles appeared from 8 dpi (Figure 6C). No IHC staining was ever detected in the mock-challenged larvae.

## Discussion

The NNV is considered a very hazardous aquatic pathogen threatening worldwide marine fish farms, with larvae and juveniles representing the most susceptible stages. To date, no effective therapy has been authorized to treat the disease and to prevent the loss of such large numbers of fish. Thus, it is critical to identify and assess potential routes of NNV entry into hatcheries, including the marine invertebrates used as starting feed for fish larval stages.

In this study, we have demonstrated the role of rotifers in transmitting NNV to sea bass larvae. Sea bass is one of the most valuable species in southern European aquaculture and is severely affected by RGNNV strains (1, 24–26). The results obtained revealed that just one administration of rotifers retaining NNV is sufficient to cause disease and 100% mortality in sea bass larvae as occurred in the bath infection, suggesting that the intake of live feed retaining NNV can trigger a VNN outbreak in a hatchery. However, the course of oral and bath infection was different, in contrast to findings by Lampou et al. (27), who reported a similar disease progression in infection *via* the skin or the esophagus. In particular, in our results, it was observable that regardless of the assay used (IHC analysis, viral titration, and viral genome quantification), orally infected individuals showed highly variable results during the first 15 dpi. This is most likely due to the different sensitivity of the applied methods, the low prevalence of positive subjects in this time frame, and the number of sampled specimens. It can also be stated that water-borne infection resulted in faster viral replication within a narrower period than by the oral infection route. The latter initially seemed to have low efficiency, resulting in asynchronous and time-lapse replication events in the larvae. Heterogeneous results shed light on the importance of the sampling size, regardless of the infection route, in order to gain a suitable representation of the population, especially during the initial phases of the infection. Despite the low oral infection rate, viral replication and shedding from infected individuals were sufficient to trigger infection in the remaining members of the population. In bath infected fish, clinical signs and histopathological findings were observed earlier than in larvae orally challenged, which might be due to the different intake of rotifers by each larva, the heterogeneity in the number of infective particles contained by each rotifer, and to the possible degradation of viral particles during the digestion process in larvae digestive tract.

The horizontal transmission routes assayed in this study demonstrated the high susceptibility to NNV of 12 dph sea bass larvae, which still do not possess a fully functional immune system (28, 29). As a matter of fact, the oral transmission indicated that viral particles contained within rotifers maintained their infectivity and virulence, showing from 15 dpi a replication kinetic comparable with that obtained in the bath infection. Rotifers (*B. plicatilis*) and the crustacean *Artemia* sp. are the most common marine invertebrates used to feed marine fish larvae. Previous studies have shown that other rotifer species (*Brachionus urceus*) can efficiently transmit white spot syndrome virus (WSSV) to crayfish (*Procambarus clarkii*) and to shrimp as *Penaeus monodon* and *Fenneropenaeus chinensis* (30–32), or act as mechanical vectors of the Covert Mortality Nodavirus (CMNV) in Pacific white shrimp (*Litopenaeus vannamei*) ponds (33). *Artemia salina* was also demonstrated to transmit NNV infective particles to Senegalese sole larvae, leading to high mortality and clinical signs similar to the bath infection (19). Both *B. plicatilis* and *Artemia salina* are filter feeders, able to internalize viruses or bacteria suspended in the water, including viral particles attached to the phytoplankton following the phytoplankton-adhesion route (30). Similar to NNV location in *Artemia* individuals (19), we identified the presence of RGNNV particles in rotifers through IFAT and IHC. Virus antigens were mainly localized in the corona and the masticatory organ (34), in the intestine lumen, and on the inner side of the cuticula. This localization could be due to the internalization of viral particles in the intestine through the masticatory organ. Once there, viral particles could pass through the peritrophic membrane, diffuse in the perivisceral fluid of the pseudocoelom, and attach to the cuticle on the inner surface. Although the interaction and attachment of NNV with rotifer cells remain unknown, WSSV was shown to adhere to rotifer cell membranes (31). In addition, the visualization of IHC staining within the rotifer body demonstrated the maintenance of NNV inside the rotifer at least up to 48 hpc. However, once NNV-exposed rotifers were placed in a virus-free environment, the presence of NNV started decreasing rapidly over time reaching a complete clearance at 6 dpc, both in terms of viable particles and detectable viral genomic RNA, suggesting that NNV accumulation into rotifers would be limited. Such evidence confirmed previous reports of viral clearance after 48 hpc and the role of some species of invertebrates as mechanical vectors in viral transmission (18).

Despite the fact that the virus is neither able to replicate nor be retained within rotifers for a long time, complete mortality was recorded in larvae orally challenged, highlighting the importance of not underestimating the role of these invertebrates in NNV transmission. In conclusion, our results indicate that NNV particles present in marine water can be transmitted to fish larvae using rotifers as mechanical vectors. Infected fish developed typical disease signs and suffered 100% mortality as in a bath infection. The final viral load was similar in fish infected by both routes, although a delay in viral replication of approximately 10 days was observed in the orally infected sea bass. In addition, it cannot be ruled out the role of phytoplankton as an intermediate vector for rotifer to larvae transmission as already experimentally demonstrated (30, 35, 36). These results highlight the importance of establishing routine controls on the live feed in order to prevent NNV horizontal transmission and to maintain the fish stock in adequate conditions for proper performance.

## Data Availability Statement

The original contributions presented in the study are included in the article/Supplementary Material, further inquiries can be directed to the corresponding author/s.

## Ethics Statement

The animal study was reviewed and approved by Italian Ministry of Health Authorization No. 1018/2020-PR.

## Author Contributions

LV-S, FP, and AM performed animal infection experiment. MA and LB performed molecular test. LV-S and AB performed virological test and IFAT. TP and EM performed histological test. IB and AT supervised the writing. AT gathered funds. All authors read and approved the final manuscript.

## Funding

The research leading to these results has received funding from the European Union's Horizon 2020 research and innovation program under Grant Agreement No. 731014 (VetBioNet project).

## Conflict of Interest

The authors declare that the research was conducted in the absence of any commercial or financial relationships that could be construed as a potential conflict of interest.

## Publisher's Note

All claims expressed in this article are solely those of the authors and do not necessarily represent those of their affiliated organizations, or those of the publisher, the editors and the reviewers. Any product that may be evaluated in this article, or claim that may be made by its manufacturer, is not guaranteed or endorsed by the publisher.
