# Supplementary material for: Role of Rotifers in Betanodavirus Transmission to European Sea Bass Larvae
Source: Front Vet Sci. 2022 Aug 3;9:932327. doi: 10.3389/fvets.2022.932327 (PMC9383259; doi:10.3389/fvets.2022.932327)
Supplement: Supplementary file 1 [file Table_1.DOCX]

**Supplementary Material S.1A and S.1B**: Systematic results of IHC staining and vacuolization in central nervous system and retina of oral challenged larvae **(1A)** *vs* bath challenged larvae **(1B)**.

| **1A. Oral challenged sea bass larvae** | | | | | | | | | | |
| --- | --- | --- | --- | --- | --- | --- | --- | --- | --- | --- |
| dpi | Total larvae on the slide (CNS) | IHC + CNS (n) | % IHC+ CNS | Vacuolated CNS (n) | % Vacuolated CNS | Total visible retinas (n) | IHC + retina (n) | % IHC+ retinas | Vacuolated retinas (n) | % Vacuolated retinas |
| 1 | 5 | 0 | 0.0 | 0 | 0.0 | 5 | 0 | 0.0 | 0 | 0.0 |
| 2 | 10 | 0 | 0.0 | 0 | 0.0 | 10 | 0 | 0.0 | 0 | 0.0 |
| 3 | 9 | 0 | 0.0 | 0 | 0.0 | 9 | 0 | 0.0 | 0 | 0.0 |
| 4 | 8 | 0 | 0.0 | 0 | 0.0 | 8 | 0 | 0.0 | 0 | 0.0 |
| 5 | 14 | 0 | 0.0 | 0 | 0.0 | 14 | 0 | 0.0 | 0 | 0.0 |
| 6 | 9 | 0 | 0.0 | 0 | 0.0 | 9 | 0 | 0.0 | 0 | 0.0 |
| 7 | 8 | 0 | 0.0 | 0 | 0.0 | 8 | 0 | 0.0 | 0 | 0.0 |
| 8 | 13 | 1 | 7.7 | 0 | 0.0 | 13 | 0 | 0.0 | 0 | 0.0 |
| 9 | 7 | 1 | 14.3 | 1 | 14.3 | 7 | 1 | 14.3 | 1 | 14.3 |
| 10 | 7 | 0 | 0.0 | 0 | 0.0 | 7 | 0 | 0.0 | 0 | 0.0 |
| 11 | 6 | 0 | 0.0 | 0 | 0.0 | 6 | 0 | 0.0 | 0 | 0.0 |
| 12 | 12 | 0 | 0.0 | 0 | 0.0 | 12 | 0 | 0.0 | 0 | 0.0 |
| 13 | 9 | 0 | 0.0 | 0 | 0.0 | 9 | 0 | 0.0 | 0 | 0.0 |
| 14 | 12 | 0 | 0.0 | 0 | 0.0 | 12 | 0 | 0.0 | 0 | 0.0 |
| 15 | 10 | 1 | 10.0 | 0 | 0.0 | 10 | 0 | 0.0 | 0 | 0.0 |
| 16 | 13 | 3 | 23.1 | 2 | 15.4 | 13 | 0 | 0.0 | 0 | 0.0 |
| 17 | 15 | 6 | 40.0 | 1 | 6.7 | 15 | 1 | 6.7 | 0 | 0.0 |
| 18 | 7 | 4 | 57.1 | 1 | 14.3 | 7 | 1 | 14.3 | 0 | 0.0 |
| 19 | 12 | 11 | 91.7 | 6 | 50.0 | 7 | 6 | 85.7 | 0 | 0.0 |
| 20 | 11 | 11 | 100.0 | 9 | 81.8 | 11 | 10 | 90.9 | 1 | 9.1 |
| 21 | 12 | 12 | 100.0 | 12 | 100.0 | 12 | 11 | 91.7 | 3 | 25.0 |
| 22 | 7 | 7 | 100.0 | 7 | 100.0 | 7 | 7 | 100.0 | 0 | 0.0 |
|  | | | | | | | | | | |
| **1B. Bath challenged sea bass larvae** | | | | | | | | | | |
| dpi | Total larvae on the slide (CNS) | IHC + CNS (n) | % IHC+ CNS | Vacuolated CNS (n) | % Vacuolated CNS | Total visible retinas (n) | IHC + retina (n) | % IHC+ retinas | Vacuolated retinas (n) | % Vacuolated retinas |
| 1 | 11 | 0 | 0.0 | 0 | 0.0 | 10 | 0 | 0.0 | 0 | 0.0 |
| 2 | 13 | 0 | 0.0 | 0 | 0.0 | 10 | 0 | 0.0 | 0 | 0.0 |
| 3 | 10 | 2 | 20.0 | 0 | 0.0 | 9 | 0 | 0.0 | 0 | 0.0 |
| 4 | 11 | 10 | 90.9 | 0 | 0.0 | 11 | 0 | 0.0 | 0 | 0.0 |
| 5 | 14 | 14 | 100.0 | 0 | 0.0 | 9 | 4 | 44.4 | 0 | 0.0 |
| 6 | 14 | 14 | 100.0 | 3 | 21.4 | 14 | 12 | 85.7 | 0 | 0.0 |
| 7 | 14 | 14 | 100.0 | 9 | 64.3 | 12 | 12 | 100.0 | 0 | 0.0 |
| 8 | 16 | 16 | 100.0 | 14 | 87.5 | 10 | 10 | 100.0 | 2 | 20.0 |
| 9 | 16 | 16 | 100.0 | 15 | 93.8 | 12 | 12 | 100.0 | 1 | 8.3 |
| 10 | 16 | 16 | 100.0 | 13 | 81.3 | 12 | 12 | 100.0 | 4 | 33.3 |
| 11 | 8 | 8 | 100.0 | 7 | 87.5 | 4 | 4 | 100.0 | 3 | 75.0 |
| 12 | 15 | 15 | 100.0 | 15 | 100.0 | 10 | 10 | 100.0 | 4 | 40.0 |
|  |  |  |  |  |  |  |  |  |  |  |
