# Supplementary material for: Role of Rotifers in Betanodavirus Transmission to European Sea Bass Larvae
Source: Front Vet Sci. 2022 Aug 3;9:932327. doi: 10.3389/fvets.2022.932327 (PMC9383259; doi:10.3389/fvets.2022.932327)
Supplement: Supplementary file 2 [file Table_2.DOCX]

**Supplementary Material S2**: LCN values of triplicates and mean ± relative standard deviation values in pooled sea bass larvae collected during oral and bath infection. Rep1. Rep2 and Rep3: biological replicates.

|  | Bath infection | | | | Oral infection | | | |
| --- | --- | --- | --- | --- | --- | --- | --- | --- |
| dpi | Rep1 | Rep2 | Rep3 | Mean ± SD | Rep1 | Rep2 | Rep3 | Mean ± SD |
| 1 | 4.00 | 3.69 | 3.84 | 3.84 ± 0.15 | 1.40 | 1.34 | 0.00 | 0.91 ± 0.79 |
| 2 | 4.84 | 5.58 | 5.13 | 5.19 ± 0.37 | 0.00 | 0.95 | 2.14 | 1.03 ± 1.07 |
| 3 | 6.45 | 6.54 | 6.60 | 6.53 ± 0.05 | 1.52 | 1.49 | 1.31 | 1.44 ± 0.11 |
| 4 | 7.70 | 7.61 | 7.65 | 7.66 ± 0.05 | 3.21 | 1.39 | 0.00 | 1.53 ± 1.61 |
| 5 | 8.74 | 8.70 | 9.15 | 8.87 ± 0.25 | 1.14 | 5.69 | 2.21 | 3.01 ± 2.37 |
| 6 | 8.82 | 9.22 | 8.59 | 8.88 ± 0.32 | 2.28 | 3.06 | 2.29 | 2.54 ± 0.45 |
| 7 | 8.96 | 9.21 | 8.98 | 9.05 ± 0.14 | 1.02 | 0.00 | 2.40 | 1.14 ± 1.20 |
| 8 | 9.11 | 9.25 | 9.10 | 9.16 ± 0.09 | 0.97 | 0.00 | 3.28 | 1.42 ± 1.68 |
| 9 | 9.10 | 8.80 | 8.97 | 8.95 ± 0.15 | 6.18 | 4.86 | 1.31 | 4.11 ± 2.51 |
| 10 | 8.73 | 9.28 | 9.10 | 9.04 ± 0.28 | 3.50 | 2.36 | 3.44 | 3.10 ± 0.64 |
| 11 | 9.31 | 9.17 | 9.05 | 9.18 ± 0.13 | 2.82 | 0.00 | 3.58 | 2.13 ± 1.88 |
| 12 | 9.15 | 8.94 | 9.01 | 9.04 ± 0.11 | 3.29 | 2.27 | 2.34 | 2.63 ± 0.57 |
| 13 |  |  |  |  | 4.75 | 3.11 | 2.62 | 3.50 ± 1.11 |
| 14 |  |  |  |  | 2.38 | 3.55 | 3.18 | 3.04 ± 0.60 |
| 15 |  |  |  |  | 5.71 | 4.64 | 3.40 | 4.58 ± 1.15 |
| 16 |  |  |  |  | 5.29 | 6.08 | 4.69 | 5.35 ± 0.70 |
| 17 |  |  |  |  | 7.13 | 6.31 | 7.26 | 6.90 ± 0.51 |
| 18 |  |  |  |  | 7.11 | 7.58 | 6.75 | 7.15 ± 0.42 |
| 19 |  |  |  |  | 7.41 | 8.17 | 8.06 | 7.88 ± 0.41 |
| 20 |  |  |  |  | 8.10 | 8.42 | 8.52 | 8.35 ± 0.22 |
| 21 |  |  |  |  | 8.27 | 8.32 | 7.96 | 8.18 ± 0.20 |
| 22 |  |  |  |  | 8.45 | 7.80 | 8.09 | 8.11 ± 0.33 |
| 23 |  |  |  |  | 7.93 | 8.04 | 8.28 | 8.08 ± 0.18 |
